# Decellularized Diaphragmatic Muscle Drives a Constructive Angiogenic Response In Vivo

**DOI:** 10.3390/ijms19051319

**Published:** 2018-04-28

**Authors:** Mario Enrique Alvarèz Fallas, Martina Piccoli, Chiara Franzin, Alberto Sgrò, Arben Dedja, Luca Urbani, Enrica Bertin, Caterina Trevisan, Piergiorgio Gamba, Alan J. Burns, Paolo De Coppi, Michela Pozzobon

**Affiliations:** 1 Stem Cells and Regenerative Medicine Lab, Fondazione Istituto di Ricerca Pediatrica Città della Speranza, Padova 35127 Italy; marioe.alvarezf@gmail.com (M.E.A.F.); m.piccoli@irpcds.org (M.P.); c.franzin@irpcds.org (C.F.); enrica.bertin@gmail.com (E.B.); trevisan-caterina@libero.it (C.T.); 2 Department of Women and Children Health, University of Padova, Padova 35100, Italy; albertosgro@gmail.com (A.S.); piergiorgio.gamba@unipd.it (P.G.); 3 Department of Cardiac, Thoracic and Vascular Sciences, University of Padova, Padova 35100, Italy; arben.dedja.pd@gmail.com; 4 Stem Cells & Regenerative Medicine Section, Developmental Biology & Cancer Programme, UCL Great Ormond Street Institute of Child Health, London WC1N 1EH, UK; urbani81@gmail.com (L.U.); a.burns@ac.ucl.uk (A.J.B.); Paolo.DeCoppi@gosh.nhs.uk (P.D.C.); 5 Department of Clinical Genetics, Erasmus Medical Centre, Wytemaweg 80 3015 CN, Rotterdam, The Netherlands

**Keywords:** skeletal muscle, tissue engineering, angiogenesis, microenvironment

## Abstract

Skeletal muscle tissue engineering (TE) aims to efficiently repair large congenital and acquired defects. Biological acellular scaffolds are considered a good tool for TE, as decellularization allows structural preservation of tissue extracellular matrix (ECM) and conservation of its unique cytokine reservoir and the ability to support angiogenesis, cell viability, and proliferation. This represents a major advantage compared to synthetic scaffolds, which can acquire these features only after modification and show limited biocompatibility. In this work, we describe the ability of a skeletal muscle acellular scaffold to promote vascularization both ex vivo and in vivo. Specifically, chicken chorioallantoic membrane assay and protein array confirmed the presence of pro-angiogenic molecules in the decellularized tissue such as HGF, VEGF, and SDF-1α. The acellular muscle was implanted in BL6/J mice both subcutaneously and ortotopically. In the first condition, the ECM-derived scaffold appeared vascularized 7 days post-implantation. When the decellularized diaphragm was ortotopically applied, newly formed blood vessels containing CD31^+^, αSMA^+^, and vWF^+^ cells were visible inside the scaffold. Systemic injection of Evans Blue proved function and perfusion of the new vessels, underlying a tissue-regenerative activation. On the contrary, the implantation of a synthetic matrix made of polytetrafluoroethylene used as control was only surrounded by vWF^+^ cells, with no cell migration inside the scaffold and clear foreign body reaction (giant cells were visible). The molecular profile and the analysis of macrophages confirmed the tendency of the synthetic scaffold to enhance inflammation instead of regeneration. In conclusion, we identified the angiogenic potential of a skeletal muscle-derived acellular scaffold and the pro-regenerative environment activated in vivo, showing clear evidence that the decellularized diaphragm is a suitable candidate for skeletal muscle tissue engineering and regeneration.

## 1. Introduction

In complex organs and tissues the vascular network, besides assuring an appropriate supply of nutrients and oxygen for regular tissue function, provides a specific interphase to allow the balance of tissue homeostasis and immune functions [1,2]. The growth of new blood vessels requires interactions between endothelial cells, soluble growth factors, and a complex network of extracellular matrix (ECM) components. For all these reasons, to repair a damaged tissue it is of paramount importance to unravel strategies that keep, and at the same time, stimulate vessels with the final aim of obtaining a functional regeneration. Thus, for a wide range of clinical applications, the ability to stimulate and control angiogenesis has a significant impact. Specifically, successful tissue engineering strategies rely on the efficiency of vascularization and subsequent tissue integration. This aspect is indeed fundamental to avoid transplant failure and, to date, experimental approaches for organ and tissue replacement have been focused on different approaches: the functionalization of natural or synthetic scaffolds with proteins or cells known to be involved in angiogenesis (in situ vascularization) [3,4], the generation of an efficient vascular network before graft implantation (pre-vascularization) [5,6]. Since angiogenesis in situ relies mainly on the host cells and pre-existing vasculature, limitations on the use of scaffolds become evident when little porous, non elastic materials and large defects are considered. Indeed, for skin wounds and small or mid-size ulcers, scaffold implantation supports spontaneous regeneration [7]. Differently, in cases of transplantation of large portions of tissue or even whole organs, the prolonged time required for vascularization of the construct through correct anastomosis with the host could result in the formation of a necrotic core or in the development of fibrotic tissue, thereby leading to graft failure [8,9,10,11]. With the aim of accelerating angiogenesis, a natural scaffold possessing similar biological and structural features to the native damaged tissue could be advantageous, since new vessels can be driven from the host tissue toward the three-dimensional scaffold [12,13,14]. Moreover, the molecules originally hidden in healthy tissue, called cryptic molecules, are released from the remodeled ECM and are known to be supportive of several biological processes [15,16,17].

As for the other tissues, in skeletal muscle the vascular network also plays a key role during remodeling and regeneration [12,13,14,15,16,17,18,19,20,21,22]. It is established that the implantation of any foreign device, from synthetic products or acellular biomaterials to even living tissue constructs, always results in its interactions with the host immune system, remodeling ECM and influencing neo-angiogenesis [23]. Immune cells, particularly macrophages, play a critical role in regulating wound healing and tissue remodeling [24]. They can indeed shift the response towards a positive outcome by means of stimulating new vases formation, matrix remodeling, and immunotolerance [25].

Recently, our group developed an acellular matrix derived from mouse diaphragmatic muscle, which was able to positively modulate immune response and led to a local proregenerative environment upon transplantation, both in healthy and diseased mouse models [26].

In this study, we characterized the angiogenic properties of a decellularized diaphragm (DD) scaffold both ex vivo and in vivo. Specifically, in vivo we have chosen two different murine models where we applied DD and a synthetic nonfunctionalized material, the expanded polytetrafluoroethylene (ePTFE) used in clinical practice, as control: the first model expects the implant under the murine back skin and for the second we used the already established orthotopic implant in healthy diaphragm [26]. Our findings not only confirm the angiogenic properties of the ECM obtained by skeletal muscle decellularization, but also underline a highly stable and prolonged angiogenic stimulus, endorsing the use of this scaffold as an alternative support for skeletal muscle defect repair.

## 2. Results

### 2.1. Diaphragm-Derived Decellularized Matrix Retains Angiogenic Potential

**Figure 1:**
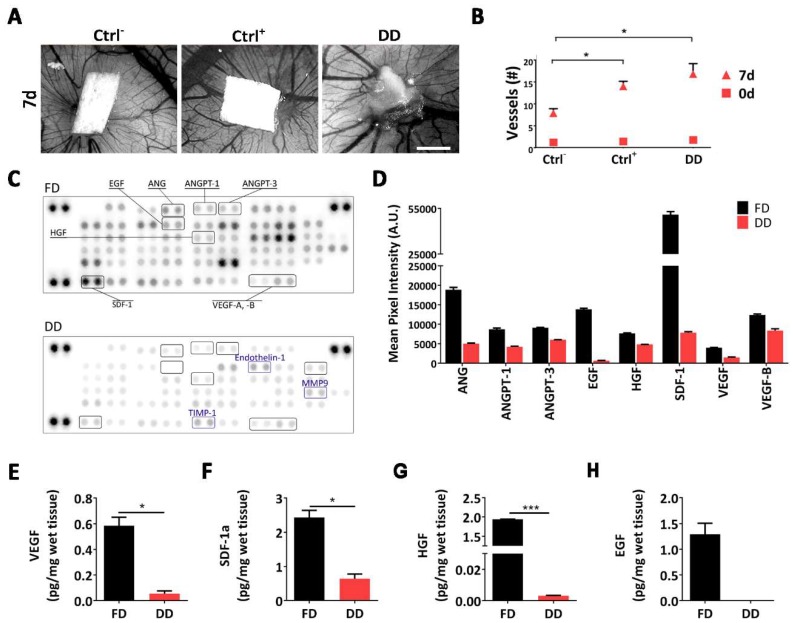
Characterization of diaphragmatic acellular scaffold angiogenic potential. (**A**) Chorion allantoic membrane (CAM) assay appearance after 7 days of incubation with negative control, positive control, and decellularized diaphragm. Scale bar = 1 mm; (**B**) Number of vessels converging towards each sample at baseline (0d) and after 7 days; vessels were counted in a blinded fashion; (**C**) Immunoblotted membrane array of fresh and decellularized tissue homogenate. 53 angiogenic factors were detected; (**D**) Pixel intensity of some of the principal angiogenic factors. Intensity was calculated using UVITEC Cambridge software (mean ± SD). Quantification of (**E**) VEGF; (**F**) SDF-1a; (**G**) HGF; and (**H**) EGF from fresh and decellularized tissue homogenate (mean ± SD). Ctrl^−^ = Negative Control (inert polyester only); Ctrl^+^ = Positive Control (polyester with VEGF); DD = Decellularized Diaphragm; FD = Fresh Diaphragm. * *p* < 0.01; *** *p* < 0.0001

Chorion allantoic membrane (CAM) assay was used to evaluate blood vessel chemo-attractive activity of DD scaffold. Samples along with positive (polyester loaded with VEGF) and negative (polyester only) controls were analyzed daily under a stereomicroscope. Seven days after implantation, neovessels were organized in a network surrounding the tissue samples (Figure 1A). Quantification of vessel growth (i.e., blood converging towards the matrix) displayed a significant increase in DD with respect to controls at the same time-point (*p* < 0.05; Figure 1B).

To both confirm CAM assay results and verify whether the DD matrix retained pro-angiogenic factors, an immune array directed against 53 proteins specifically involved in mouse angiogenesis was performed. Most of the analyzed proteins were detected in the decellularized tissue (Figure 1C,D), although with lower intensity compared to fresh tissue. In particular, specific angiogenic cytokines (e.g., VEGF and Angiopoietins), matrikines, and enzymes necessary for endothelialization (e.g., endothelin or metalloproteases such as MMP9) were detected. Furthermore, for VEGF, SDF-1α, HGF, and EGF, key factors regulating vasculogenesis and angiogenesis, we have also measured the amount retained in the decellularized tissue via ELISA test. Despite a significant decrease, 3 out of 4 factors were still present in a detectable amount in the tissue after decellularization (VEGF: 0.580 ± 0.06 pg/mg in FD, 0.068 ± 0.02 pg/mg in DD; SDF-1α: 2.432 ± 0.204 pg/mg in FD, 0.642 ± 0.13 pg/mg in DD; HGF: 1.939 ± 0.02 pg/mg in FD, 0.003 ± 0.0001 pg/mg in DD. Figure 1E–H).

### 2.2. Cell-Scaffold Interaction

**Figure 2:**
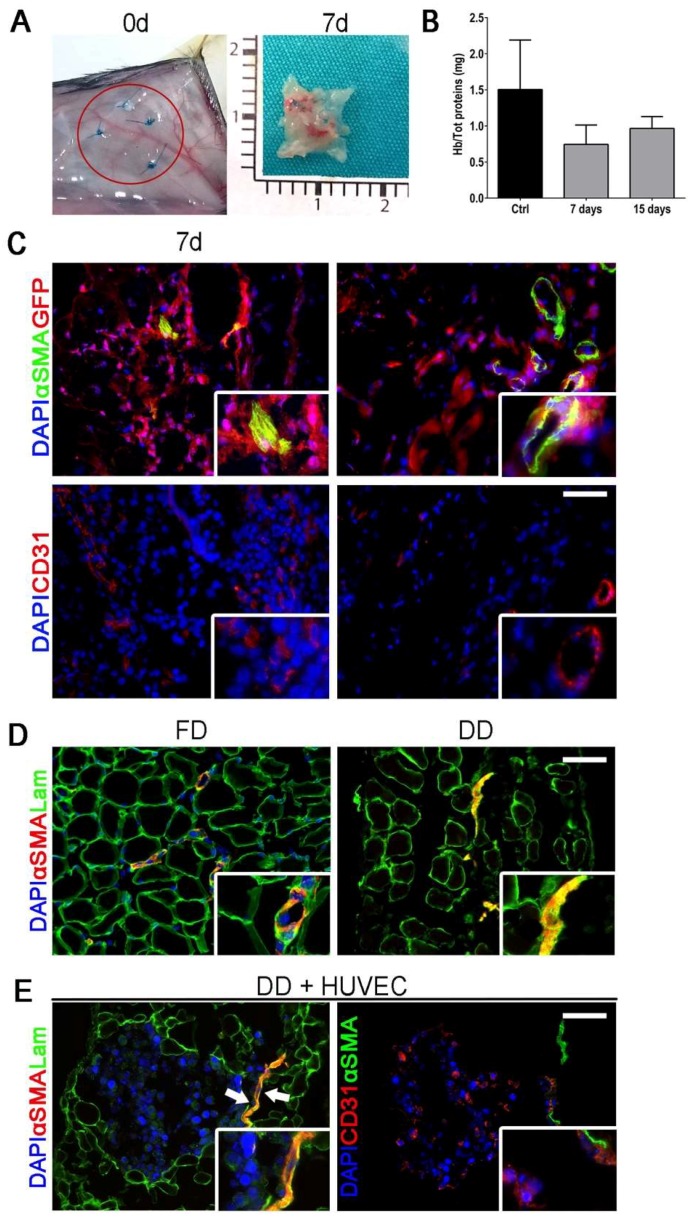
Scaffold subcutaneous implantation and vessel after DET treatment. (**A**) Macroscopic appearance of the implanted DD at day 0 (red circle) and of DD explanted after 7 and 15 days of implantation; (**B**) Quantification of hemoglobin over total protein content in control (skin) and DD after 7 and 15 days of implantation (mean ± SD); (**C**) Immunofluorescence staining against αSMA and GFP performed in explanted DD after 7 and 15 days from implantation (upper panel) and CD31 (lower panel); nuclei were counterstained with DAPI; (**D**) Immunofluorescence staining against αSMA and Laminin performed in fresh diaphragm (FD) and DD; nuclei were counterstained with DAPI; (**E**) Immunofluorescence staining against αSMA and Laminin or CD31 performed in DD after 48 h of incubation with HUVECs; nuclei were counterstained with DAPI; Scale bar = 100 µm.

Once the attraction of new vessels was confirmed, their origin was investigated to identify whether neovascularization was due to new vessel formation, or if the pre-existing vessels were recellularized by host cells. To this aim, we performed implantation in GFP^+^ mice for host cell-tracking (Figure 2A).

Transplanted patches appeared vascularized at 7 days (Figure 2B), confirming CAM assay results. The amount of hemoglobin (Hb) quantified on the harvested DD patches, corroborated the previous data with a tendency of Hb to increment with time, suggesting also a functional angiogenesis with perfused vessels inside the applied patch (Figure 2C). Immunofluorescence performed on DD excised 7 days post implant revealed the presence of αSMA^+^ vessels with uneven appearance and morphology (Figure 2D). Among all, some of them were αSMA^+^ but did not have any GFP^+^ cell inside, indicating the presence of empty vessels (Figure 2D, 7-day right inset). Fifteen days after implant, the majority of the analyzed vessels had a uniform shape and dimension as pointed out by the presence of GFP^+^ cells inside the vessel structure (Figure 2D, 15-day right inset). To confirm the hypothesis that endothelial cells recolonized leftover vascular structures in the transplanted matrix, HUVEC were seeded in vitro on top of the DD and 48 h later their migration was analyzed. The vast majority of pre-existing vascular structures was recognized by HUVEC (CD31^+^ cells), confirming the influence of old vasculature structure on patch angiogenic features and host endothelial cell behavior (Figure 2D,E)

### 2.3. Angiogenic Response to Orthotopic Transplantation of DD vs. ePTFE

**Figure 3:**
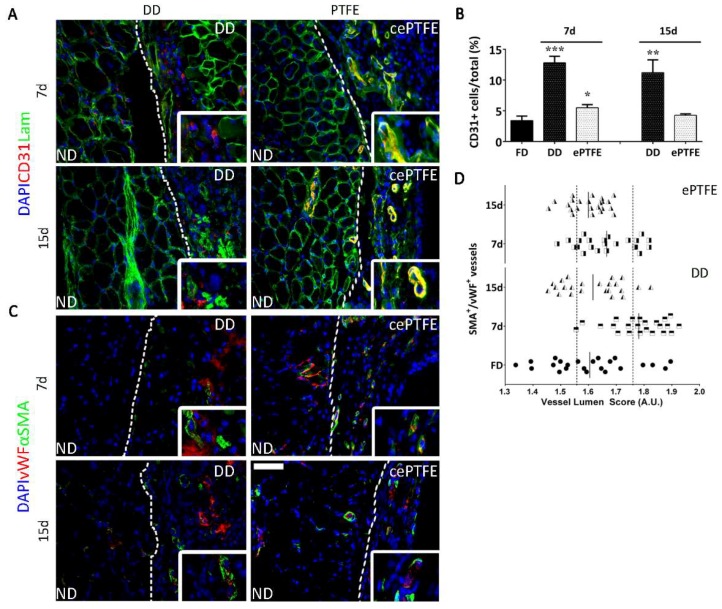
Comparison of angiogenic response after orthotropic implantation of DD vs. ePTFE. White line marks the normal diaphragm (ND) versus the patch (decellularized diaphragm –DD- or plastic –ePTFE- or capsule expanded –cePTFE-) (**A**) Immunofluorescence staining against CD31 and Laminin performed in explanted DD and ePTFE after 7 and 15 days from implantation; nuclei were counterstained with DAPI; (**B**) Quantification of CD31^+^ cells over total cell content in explanted DD and ePTFE after 7 and 15 days from implantation (mean ± SD); (**C**) Immunofluorescence staining against vWF and αSMA performed in explanted DD and ePTFE after 7 and 15 days from implantation; nuclei were counterstained with DAPI; (**D**) αSMA^+^/vWF^+^ vessels dimension distribution calculated in fresh tissue, DD and ePTFE after 7 and 15 days from implantation. Symbol legend. Black dots: vessel dimension of the fresh diaphragm (control); black and white squares: vessel dimension 7 days post implant; black and white triangles: vessel dimension 15 days post implant. Scale bar = 100 µm. * *p* < 0.01; ** *p* < 0.001; *** *p* < 0.0001.

**Figure 4:**
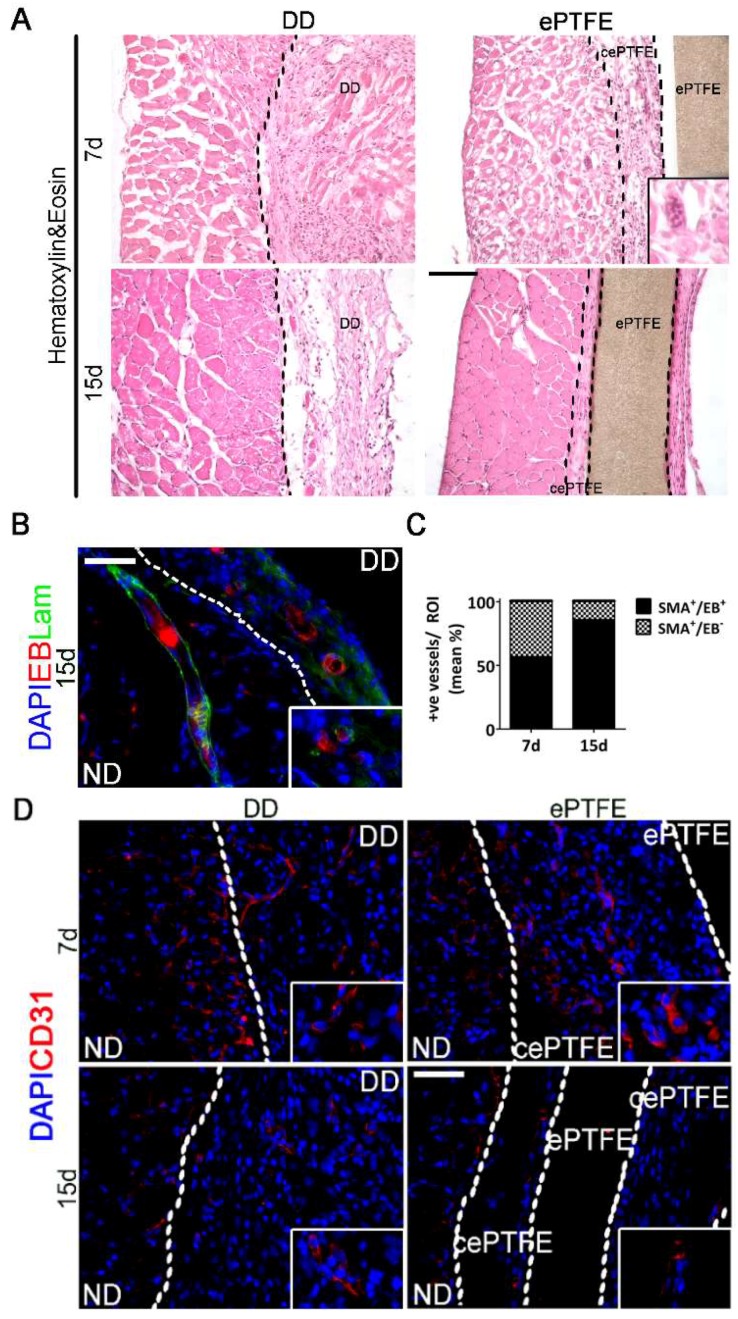
Detection of vases in DD vs. ePTFE after orthotropic implantation. White or black line marks the normal diaphragm (ND) versus the patch (decellularized diaphragm –DD- or plastic –ePTFE- or capsule expanded –cePTFE-); (**A**) Histological appearance of DD and ePTFE-implanted diaphragm. Haematoxylin&Eosin staining performed in DD and ePTFE after 7 and 15 days from implantation. No vessels inside the ePTFE; ePTFE 7 days inlet displays a foreign body giant cell. Scale bar = 100 µm; (**B**) Functional vessels after 15 days of DD implantation. Immunofluorescence staining against Laminin performed on DD-implanted diaphragm after 15 days of implantation; Evans Blue dye is autofluorescent in the red spectrum; nuclei were counterstained with DAPI; (**C**) Quantification of αSMA^+^/EB^+^ and αSMA^+^/EB^−^ vessels content in DD after 7 and 15 days from implantation (mean %); (**D**) Immunofluorescent staining against CD31^+^ cells performed n DD- and ePTFE-implanted diaphragms 7 and 15 days post implantation. Scale bar = 100 µm.

Having demonstrated formation of functional vasculature, we evaluated DD angiogenic properties by orthotopic implantation (Figure 3). Patches were applied on host diaphragm, in order to challenge the DD with a physiological environment, without any local injury. We compared the angiogenic effect exerted by our scaffold with that of ePTFE, a prosthetic patch already used in clinic [27]. From the histological point of view, the H & E staining showed that the biological DD underwent remodeling coherently to the outcome seen during the previous characterization [26], while ePTFE was encapsulated by a thick cellular layer, suggesting a beginning of foreign body reaction (Figure 4A).

Concerning endothelial cell presence, CD31^+^ cells migrated from the native diaphragm (ND) in the applied scaffold after 7 and 15 days post implantation (Figure 3A). In both the analyzed time points endothelial cells increased significantly (*p* < 0.0001) in DD samples compared to a fresh untreated diaphragm (Figure 3B). Furthermore, although CD31^+^ cells could be detected also in the fibrotic capsule surrounding the implanted ePTFE, their number was not significantly different from a basal untreated condition (Figure 3B). These results suggest a stronger angiogenic effect of the applied naturally-derived ECM with respect to prosthetic material.

We co-stained αSMA as a vessels perimetric marker, together with vWF to confirm both vessel function and luminal size, which allowed the measurement of the vessel size (Figure 3C,D). In the DD implants, at 7 days postsurgery the distribution was between small and mid-size vessels (from 2.0 to 1.75, and from 1.75 to 1.55, respectively), then after 15 days it resembled a physiological condition (vessel diameter from 1.55 to lower values) (Figure 3D). On the contrary, at the interface between ePTFE and ND, vessels had a distribution similar to native condition 7 days postsurgery, whereas middle-size vessels appeared at the later time point (Figure 3D).

To confirm the presence of functioning and perfused vessels highlighted previously by the co-expression of αSMA and vWF inside the applied DD, Evans Blue dye was systemically injected. Red color stain was detected in the lumen of αSMA^+^ vessels at both time points. Moreover, the proportion of nonperfused αSMA^+^ vessels diminished through the time points, indicating an increase of functional vessels (Figure 4B,C).

### 2.4. Molecular Profiling of Host-Scaffold Interaction

**Figure 5:**
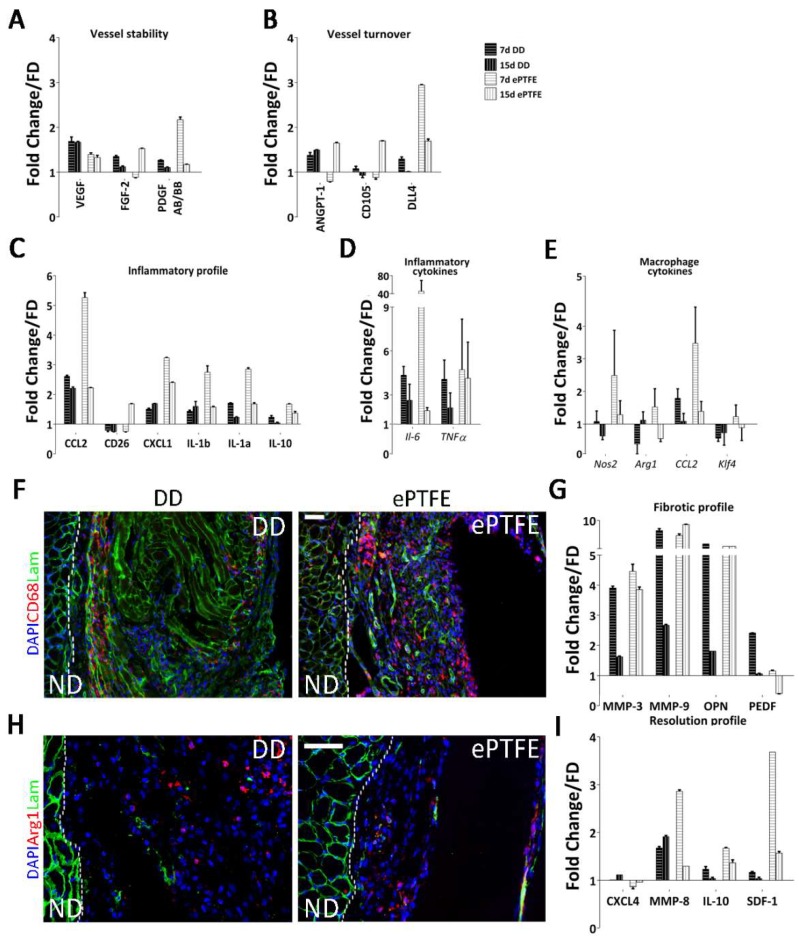
Molecular response and immunoreaction elicited by DD vs. ePTFE. White or black dot line marks the normal diaphragm (ND) versus the patch (decellularized diaphragm –DD- or plastic –ePTFE-) (**A**,**B**) Quantification of angiogenic factors from DD and ePTFE-implanted diaphragms after 7 and 15 days compared to native tissue (set at 1), grouped by the processes involved (mean ± SD); (**C**) Quantification of *Il-6* and TNF*α* mRNA extracted from DD and ePTFE after 7 and 15 days from implantation, compared to native tissue (set at 1) (mean ± SD); (**D**) Quantification of factors involved in both angiogenic and immunomodulatory processes from DD and ePTFE-implanted diaphragms after 7 and 15 days, compared to native tissue (set at 1) (mean ± SD); (**E**) Quantification of *Nos2*, *ArgI*, *CCl2* and *Klf4* mRNA extracted from DD and ePTFE after 7 and 15 days from implantation, compared to native tissue (set at 1) (mean ± SD); (**F**) Immunofluorescence staining against pan-macrophages CD68 antigen performed in DD and ePTFE implanted diaphragms after 15 days; (**G**) Quantification of factors involved in both angiogenic and fibrosis processes from DD and ePTFE-implanted diaphragms after 7 and 15 days, compared to native tissue (set at 1) (mean ± SD); (**H**) Immunofluorescence staining against M2 macrophages Arginase I antigen performed in DD and ePTFE-implanted diaphragms after 15 days; (**I**) Quantification of factors involved in both angiogenic and reconstructive processes from DD and ePTFE-implanted diaphragms after 7 and 15 days, compared to native tissue (set at 1) (mean ± SD); Scale bar = 100 µm.

The general pattern of the array data displayed by ePTFE vs. DD implants reflected the difference between the two materials in terms of properties and exerted tissue response. To aid data interpretation, proteins of interest were plotted under different categories according to the function or involvement in angiogenesis. Seven days after surgery, protein quantification in ePTFE implants displayed an abrupt increase compared to DD, supporting the rapid vessel constitution seen by immunofluorescence (Figure 4D). Specifically, of the three proteins commonly associated with neoangiogenesis, VEGF, FGF2, and PDGF-BB, only FGF2 had a different behavior when comparing the two implanted materials (Figure 5A), increasing in the ePTFE-treated diaphragms after 15 days. Regarding proteins associated with vessel turnover, Angiopoietin-1 (Angpt-1) raised in both cases, CD105 decreased in DD-implanted muscles while increasing in ePTFE implants, and Dll4 decreased towards the baseline, returning to fresh diaphragm (FD) values particularly after 15 days of DD implantation (Figure 5B). The difference among the stimuli driving angiogenesis in the two implants was confirmed by the mRNA expression of *Flt1* (VEGFR1), which appears to be progressively down- or upregulated after DD or ePTFE implantation, respectively.

In both treated samples, inflammation-associated proteins seem to indicate the persistence of an inflammatory state (Figure 5D), since Il-1a and Il-1b lingered overexpressed. Interestingly, the mRNA levels of TNFα remained considerably high after 15 days in the ePTFE-implanted diaphragms (Figure 5C). On the contrary, proteins associated with a profibrotic and proangiogenic response (Il-10, MMP-9, OPN) or with an antifibrotic and antiangiogenic effect (CXC4, MMP-8, PEDF) were expressed differently upon stimulation of the two materials at both time points. Specifically, in the ePTFE-treated samples Il-10, MMP-9, and OPN were upregulated also at 15 days, whereas antifibrotic proteins such as MMP-8 and PEDF were downregulated at the same time point, suggesting a continuous stimulation through a foreign body reaction. On the contrary, in DD-treated diaphragm MMP-9 and OPN decreased during the time points, and at the same time, MMP-8 and CXCL4 were more expressed, with PEDF highly present at the early time point (Figure 5I). In addition, CD26, a protein with a demonstrated pathogenic role in development of fibrosis of various organs, is differently regulated in DD and ePTFE with an important overexpression in the latter after 15 days (Figure 5C).

Lastly, although tissue regeneration is generally associated with M2 type macrophages, it is known that a balance response among M1 and M2 phenotype is essential for a constructive remodelling, while excess M2 can result in fibrosis. Gene expression of *Nos2* (commonly associated with M1), *Arg1*, and *Klf4* (both associated with the shift toward M2) confirmed our previous results with DD being able to stimulate a balanced shift from M1 to M2. ePTFE-implanted muscles expressed the three markers simultaneously, suggesting a copresence of the two different polarization states (Figure 5E,F,H).

## 3. Discussion

Angiogenesis remodels and extends the pre-existing vascular tree forming new capillaries [28] and this process in the context of tissue regeneration is crucial to modulate the response towards a proregenerative environment [29,30].

This work was specifically focused on defining the angiogenic properties of DD following previous studies on the murine model that already demonstrated several proregenerative effects after in vivo transplantation [26]. At first, we started by characterizing the angiogenic profile of our DD scaffold. Since it is established that the ECM provides both a mechanical support and a molecular reservoir able to influence cell behavior [31,32], it was hypothesized that the presence of molecules regulating vessel formation could be the trigger accounting for CAM result. This hypothesis was supported by several studies which demonstrated the preservation of the ECM components after decellularization [33,34], also by the means of bioactive molecules [35,36,37]. Using a specific panel against 53 molecules involved in mouse angiogenesis, we more deeply characterized the reservoir quality of our scaffold. Almost all the spots in the prepatterned mouse angiogenesis array developed a signal, indicating that most of the essayed angiogenesis-involved molecules were still present and detectable after decellularization. Focusing on the quantification of four specific cytokines, we were able to detect VEGF, known to be the master regulatory factor for angiogenesis [38], HGF, involved in both angiogenesis and skeletal muscle growth/repair [39], and SDF-1a, linked not only to angiogenesis but also to other proregenerative processes (e.g., chemotaxis of immune cells) [40]. Considering these data, it was clear that the angiogenic potential persists and is effective despite reduction in the ECM reservoir. The presence of almost all of the array proteins can be advantageous not only because the scaffold does not necessarily require preconditioning [41], but also because a desired effect (angiogenesis, for example) could be further enhanced if a chosen factor is additionally loaded. In this respect, quantification of the cytokines retained by the DD could be the initial step for a tunable control of this process.

Next, subcutaneous implant [42] was used as common method to evaluate angiogenic potential of the biological scaffold. In an allogeneic setting, GFP^+^ mouse was chosen as recipient to mark and recognize the host cells and vessels. At both analyzed time points, GFP^+^/αSMA^+^ vessels indicated a complete integration with the host vasculature. Hemoglobin quantification in the explanted tissue highlighted the functionality of these vessels and blood flow activity. Interestingly, decellularization preserved the innermost (as intima was depleted) connective layer, but also the hollow structure of the media, since αSMA^+^ vessels could be found in the DD. We decided to use endothelial-like cells such as HUVEC [43] as a tool to investigate whether the vessels preserved after decellularization could be recognized as vascular mold. We demonstrated that HUVEC migrated towards the vessel structures considered as their natural location. This result might be a clue of what happens in vivo when cells get in touch with ECM; however, to really understand the process further, studies are required.

Although the use of synthetic materials is common practice in other areas of medicine, several products, from both xeno and allogeneic origin, have been already commercialized [30], while others, both unseeded or seeded with cells, are currently in clinical use (bladder, urethra, and trachea [44,45,46]. Organs with a high modular complexity, such as the heart, lung, and liver [47,48,49], are still undergoing preclinical studies, as well as skeletal muscle [50,51]. With the orthotropic scaffold implantation, our group already proved the ability of the muscle-derived ECM to exert a proregenerative effect. Here, in line with the findings on decellularized tissue advantages over synthetic materials [52], we showed that the regeneration ability was due to the solid angiogenic properties of DD underlying strong differences between the inert ePTFE, one of the gold standard materials in clinical settings [53]. After histological analyses, DD implants attracted vessels from the host while modulating its response towards a constructive environment. On the contrary, the ePTFE seemed to have elicited an abrupt response, which after 15 days appeared to have stabilized. Recipient CD31^+^ cells could be detected in the ECM of the decellularized scaffold, as well as functional and pre-existing vessels. Presence of functional αSMA^+^/vWf^+^ vases, as well as retention of the Evans Blue dye [54], indicated the constitution of both epithelium and blood flow. ePTFE implants on one hand exerted an effect in which CD31^+^ cells were present only inside functional vessels, while on the other hand the rapidly elicited angiogenesis appeared to be simultaneously regressing, as part of a chronic turnover process. Indeed, while distribution among the size of the vessels found in DD-implanted animals ultimately resembled the natural environment with dis-homogeneous vessel dimensions (from small to big), the synthetic material stabilized on middle size vase dimensions. It is known that the prolonged contact of any device, either synthetic or biological, results in activation of angiogenesis during the cascade of events involved in the foreign body reaction [24]. Nevertheless, differences through the analyzed time points highlighted the properties of the biological scaffold in modulating the host response towards a functional remodeling. The response to the ePTFE indeed confirmed the behavior seen in a classic foreign body reaction [55], in which the host enhances angiogenesis to efficiently deliver immune cells [56] and increases the local angiogenesis (high level of FGF2 and PDGF-BB after 7 days) aiming only at restoring physiological conditions rather than constituting a proregenerative environment (Figure 4, ePTFE bars). Instead, implanted DD was recognized as tissue to be remodeled and reabsorbed, stimulating a regenerative functional angiogenesis, as demonstrated by the comparable expression of FGF2 and PDGF-BB (Figure 4, DD bars) [57]. The increased downregulation of VEGFr1 upon DD implantation, which conversely seems to be trending towards restoration in ePTFE-implanted muscles, could be a further indication of the difference between the angiogenic stimuli. A decrease in VEGFr1 could suggest either an increase in vascular sprouting or a decrease in vessel regression [58].

The two different responses achieved by DD and ePTFE patches could be appreciated also by the gene expression results. In general, the protein pattern displayed by ePTFE implants showed an intense increase after 7 days compared to DD, but in both cases for many proteins it trended similarly towards normal levels (healthy diaphragm line), suggesting that, although different, some extent of overlap had occurred in the responses. The overall mRNA and protein level (i.e., Il-6, TNFα and Il-1β, Il-10, Ccl2) were coherent to results previously seen with these two types of materials [59].

Considering the immunomodulation of the innate immune system [60], it is now established that constructive remodeling requires a fine-tuned balance in immune system cell intervention, not only to avoid rejection of the graft, but also to avoid the establishment of a chronic response [61,62]. In particular, macrophages were found to be of paramount significance, being a heterogeneous population influencing inflammation, angiogenesis, and fibrosis, both directly and indirectly [63,64]. In this respect, DD implants displayed a balanced presence of cytokines and chemokines (e.g., FGF2, CXCL4, Il-10, MMP-3, MMP-8, MMP-9, PEDF) known to be up- or downregulated by cells of the immune system to keep the physiological equilibrium as already shown [26] and involved in the remodeling of ECM, which can in turn be supportive of angiogenesis [65,66]. In this context, Real Time PCR confirmed previous results of a specific time course in macrophage polarization, from M1 to M2, in DD-transplanted animals, while ePTFE triggered an abrupt increase in the mRNA expression of *Il-6*, *TNFα*, *Nos2*, and *Ccl2*, commonly associated with inflammatory cells. The overall profile suggested that differently polarized macrophages were present simultaneously, probably due to the management of the fibrotic capsule surrounding the foreign material [67,68,69,70,71,72].

With the synthetic material, the lack of a balanced progression ultimately resulted in a nonconstructive response where angiogenesis was not consistent.

Understanding construct–host interactions is essential to avoid any adverse effect by both modulating foreign body reaction (i.e., graft vs. host) and enhancing regeneration (i.e., functional integration). Decellularized scaffolds represent a promising tool for tissue engineering. However, improvement in the development of complex organ decellularization protocols is needed to address issues that are still unanswered, such as complete xenogeneic antigen depletion, sterilization strategies to avoid pathogen transmission, and costs and availability for scale-up and large-scale production. Nevertheless, DD proved to be a promising material, with both pro-angiogenic potential and immunomodulatory properties. Precisely, the ability to direct the immune system was reflected in the foreign body reaction, which was driven towards (1) a constructive remodeling, (2) the induction of the myogenic program, (3) the formation of stable vessels, and (4) the ultimate reabsorption of the scaffold into a restoration of normal condition.

## 4. Materials and Methods

### 4.1. Animals

All surgical procedures and animal husbandry were carried out in accordance with the University of Padua’s Animal care and Use Committee (CEASA, protocol number 67/2011 approved on 21 September 2011). Eight 12-week-old male and female C57BL/6J mice (B6) were used as both donors (diaphragms for scaffolds generation) and recipients (orthotropic implantation). Twelve-week-old male and female B6 UBC-GFP mice (GFP^+^) were used as recipients for subcutaneous implantation. For the experiments carried out in the UK, all surgical procedures and animal husbandry were carried out in accordance with UK Home Office guidelines under the Animals (Scientific Procedures) Act 1986 and the Local Ethics Committee.

### 4.2. Diaphragm Decellularization

Twenty four diaphragms, harvested with the whole rib cage, were washed in sterile phosphate buffered saline (PBS) and either used as fresh diaphragm (FD) controls, or immediately treated with three cycles of a detergent-enzymatic treatment protocol as previously described [26]. Each cycle was composed of deionized water at 4 °C for 24 h, 4% sodium deoxycholate (Sigma, Milan, Italy) at room temperature (RT) for 4 h and 2000 kU DNase-I (Sigma, Milan, Italy) in 1 M NaCl (Sigma, Milan, Italy) at RT for 3 h. At the end of the third cycle, samples (DD) were left in milliQ water for a final washing step of 72 h, after which they were immediately used or preserved at 4 °C in PBS supplemented with 1% Penicillin/Streptomycin (PBS-P/S).

### 4.3. Chicken Chorioallantoic Membrane Assay

CAM assay was performed as previously described [73]. Fertilized chicken eggs (Henry Stewart and Co. 6 for negative control and 13 for the experimental conditions) were incubated at 37 °C and constant humidity. At 3 days of incubation, an oval window of approximately 3 cm in diameter was cut into the shell with small dissecting scissors to reveal the embryo and CAM vessels. The window was sealed with tape and the eggs were returned to the incubator for a further 5 days. At day 8 of incubation, 1 mm diameter acellular matrices were placed on the CAM between branches of the blood vessels. Polyester sections (ePTFE) soaked overnight either in PBS or in 200 ng/mL VEGF in PBS were used as negative and positive controls, respectively. Eggs with patches (acellular biological matrices and ePTFE) were examined daily until 7 days after placement wherein they were photographed in ovo with a stereomicroscope equipped with a Camera System (Leica, Italy) to quantify the blood vessels surrounding the matrices. The number of blood vessels converging towards the placed tissues was counted blindly by assessors, with the mean of the counts being considered.

### 4.4. Proteome Profiler Angiogenesis Array

Samples designated for protein array (5 for each group) were snap frozen and stored at −80 °C until the beginning of the assay. Before start, tissue was thawed and lysed by mechanical homogenization, and the resulting protein solution was collected in PBS with protease inhibitors (10 μg/mL Aprotinin, 10 μg/mL Leupeptin, and 10 μg/mL Pepstatin, all Sigma-Aldrich (Milan, Italy). Nonoperated B6 diaphragms served as control (FD). Three tissue homogenates for each sample (FD, DD, implanted diaphragms after 7 and 15 days with DD or ePTFE) were pooled for analysis and concentration of each pool was determined with BCA protein assay kit (Pierce, Thermo Fisher Scientific, Waltham, MA, USA, performed according to manufacturers’ instructions). Proteome profiler mouse angiogenesis array ARY015 (R&D Systems, Milan, Italy) was used according to manufacturers’ instructions. Luminescence acquisition and quantification of the pixel density of each spot was determined with Alliance (UVITEC, Cambridge, UK). For data analysis, average background signal (negative control spots) was subtracted from protein spots signal, which was subsequently normalized to positive control spots and related to signals detected from FD pool (Fold change/FD).

### 4.5. ELISA Test

Samples designated for ELISA test (5 for each group) were snap frozen and stored at −80 °C until the beginning of the assay. Before start, tissue was thawed, lysed by mechanical homogenization, and immediately assayed. Nonoperated B6 diaphragms served as control. Three decellularized diaphragm quarters (from different samples) were pooled and used for each ELISA. Quantikine for mouse VEGF, HGF, EGF, and SDF-1a (R&D systems) were carried out according to manufacturers’ instructions. Luminescence acquisition and sample quantification was performed using SpectraMax Plus 384 (Molecular devices, San Jose, CA, USA).

### 4.6. Subcutaneous Implantation

Mice were gently handled in general anesthesia with O_2_ and 1.5–2.0% isoflurane (Forane, Merial, Padova, Italy) inhalation. While in procumbent position, a medial incision was performed on the back of the mouse allowing the skin to be gently detached from the underlying fascia. For each animal, up to three portions (0.7 × 0.7 cm each) of acellular scaffold (5 pieces from different animals and for each time point) were positioned and fixed to the skin side with a Prolene 7/0 suture. Skin was then closed with a Prolene 6/0 suture and animal left to recover under a heating lamp. Mice were euthanized by cervical dislocation at 7 and 15 days post-implantation.

### 4.7. Hemoglobin Quantification

For hemoglobin quantification, DD only were carefully dissected from the host skin to prevent host tissue contamination. Drabkin’s reagent lyses red blood cells and oxidizes all forms of hemoglobin (Hb), [74]. Samples were homogenized and diluted 1⁄1000 with Drabkin’s reagent (Sigma) so that the final HiCN concentration fell within the range of the calibration curve (0–0Æ8 g/L HiCN) produced using purchased hemoglobin (Sigma). After mixing, samples and standards were incubated at RT for 30 min, protected from light. Absorbance was read at 550 nm with SpectraMax Plus 384 and the Hb concentration of each sample was calculated from the linear equation of the calibration curve.

### 4.8. Evans Blue Injection

Evans Blue (Sigma) was diluted to 0.5% in 0.9% NaCl solution. Two hundred µL of the prepared solution was injected via the tail vein and left diffusing in the bloodstream for 30 min, before animal euthanasia. To avoid loss of the dye, diaphragm muscles were fixed right after harvesting with 0.25% Glutaraldehyde (Sigma) in PBS. The procedure was performed only in DD-implanted animals and a total of 5 for each time point were essayed.

### 4.9. Human Umbilical Vein Endothelial Cells Culture and Seeding

Human umbilical vein endothelial cells (HUVEC, PromoCell) were expanded in endothelial medium (PromoCell) and used after passage 2 or 3. For the in vitro experiment of seeding HUVEC on the top of the decellularized ECM, cells were detached from the culture plate using 0.025% Trypsin-EDTA (Invitrogen, Carlsbad, CA, USA) and counted, aiming to obtain a concentration of 5 × 10^5^ cells/15 µL, which was the amount distributed on each scaffold (empty of other cells). Samples were finally left in culture for 48 h and were subsequently fixed and analyzed (5 samples).

### 4.10. Orthotopic Implantation

Surgical procedure was carried out as previously described [26]. Briefly, while placed in supine position with its caudal part towards the operator, a medial incision was performed in the abdomen of the mouse. To visualize the diaphragm, liver and stomach were then gently moved on the right downward part of the abdomen with the help of a sterile gauze soaked in warm saline solution. DD or ePTFE patches (0.7 × 0.7 cm each) were fixed on the left side of the native diaphragm with 4 Prolene 8/0 stitches. The abdominal wall was closed in two layers with 5/0 running suture and the animals were left to wake up under a heating lamp. Mice were euthanized by cervical dislocation at 7 and 15 days post-implantation (8 mice for each time point).

### 4.11. Histological Stain and Immunofluorescence

Frozen sections (thickness of 6–8 µm) were stained with HE kit for rapid frozen section (Bio-Optica, Milano, Italy) under the manufacturer’s instruction. For immunofluorescence analyses, sections were permeabilized with 0.5% Triton X-100 (10 min), blocked with 5% Horse serum/5% Goat serum (30 min) and incubated with primary antibodies overnight at 4 °C. Slides were then incubated with secondary antibodies Alexa Fluor-conjugated. Antibodies used are listed in Table S1. Nuclei were counterstained with fluorescent mounting medium plus 100 ng/mL 4′,6-diamidino-2-phenylindole (DAPI) (Sigma-Aldrich). For each count performed, *n* = 8 random pictures were collected and analyzed.

### 4.12. Vessel Size Analysis

Eight images per group (fresh, transplanted after 7 days, and 15 days) were analyzed to examine the difference in vessel dimension. To this aim, for each image the perimeter and the area of alpha smooth muscle actin (αSMA) and von Willebrand Factor (Vwf) double positive vessels were calculated and their proportion was analytically obtained by means of the fractal dimension index (FRAC, http://www.umass.edu/landeco/research/fragstats/documents/Metrics/Shape%20Metrics/Metrics/P9%20-%20FRAC.htm). The formula is commonly used to calculate the proportion of area and perimeter in irregular shape surfaces. Subsequently, the shape data expressed as vessel lumen were collected and the groups compared.

### 4.13. Real Time PCR

Total RNA was extracted using RNeasy Plus Mini kit (QIAGEN GmbH, Milan Italy) following the supplier’s instructions. RNA was quantified with an ND-2000 spectrophotometer and 1 µg total was reverse transcripted with SuperScript II and related products (all from Life Technologies, Milan, Italy) in a 20 µL reaction. Real-time PCR reactions were performed using a LightCycler II (Roche, Milan, Italy). Reactions were carried out in triplicate using 4 µL of FASTSTART SYBR GREEN MASTER (Roche) and 2 µL of primers mix Forward and Reverse (final concentration, 300/300 nM) in a final volume of 20 µL. Serial dilutions of a positive control sample were used to create a standard curve for the relative quantification. The amount of each mRNA was normalized over the expression of β2-microglobulin. Primer sequences used are listed in Table S2.

### 4.14. Statistical Analysis

Image-based counts and measurements were performed with Fiji 30 or alternatively with Imago 1 (Mayachitra, Santa Barbara, CA USA). For each analysis, at least five random pictures were used for data output. All graphs displayed were produced with GraphPad software (GraphPad Software Inc, CA, USA) 5 or 6. Data are expressed as means ± SEM. Statistical significance was determined using an equal-variance Student’s t-test for qRT-PCR analyses (*t*-test was performed between: DD 7d vs. DD 15d, DD 7d vs. ePTFE 7d, DD 15d vs. ePTFE 15d, ePTFE 7d vs. ePTFE 15d). A p value below 0.05 was considered to be statistically significant. See Table S3.

## 5. Conclusions

Summarizing, to understand and make reproducible tissue regeneration approaches, it is of paramount importance to deepen the investigation of the interactions between scaffold and host, unveiling the specific players from either the cellular, mechanical, or molecular side. In this study we used a tailored scaffold closely resembling the tissue of origin, proving that angiogenic and, consequently, immunomodulatory properties persist after decellularization and may lead to an efficient regeneration process [75,76]. These characteristics make our DD a promising scaffold to be implemented for clinical purposes in tissue engineering-based approaches.

## Supplementary Materials

Supplementary materials can be found at http://www.mdpi.com/1422-0067/19/5/1319/s1.

## Abbreviations

DD	Decellularized diaphragm
ePTFE	Expanded polytetrafluoroethylene
cePTFE	Capsule Expanded polytetrafluoroethylene
ECM	Extracellular matrix
CAM	Chorion allantoic membrane
ND	Native diaphragm
