# Supplementary material for: Decellularized Diaphragmatic Muscle Drives a Constructive Angiogenic Response In Vivo
Source: Int J Mol Sci. 2018 Apr 28;19(5):1319. doi: 10.3390/ijms19051319 (PMC5983670; doi:10.3390/ijms19051319)
Supplement: Supplementary file 1 [file ijms-19-01319-s001.pdf]

International Journal of *Molecular Sciences*

MDPI

Article

Decellularized diaphragmatic muscle drives a constructive angiogenic response in vivo.

Mario Enrique Alvarèz Fallas[1,2], Martina Piccoli[1], Chiara Franzin[1], Alberto Sgrò[2], Arben Dedja[3], Luca Urbani[4], Enrica Bertin[1], Caterina Trevisan[1,2], Piergiorgio Gamba[2], Alan J. Burns[4,5], Paolo De Coppi[4], Michela Pozzobon[1,2*]

[1] Stem Cells and Regenerative Medicine Lab, Fondazione Istituto di Ricerca Pediatrica Città della Speranza, Padova, Italy

[2] Department of Women and Children Health, University of Padova, Padova, Italy

[3] Department of Cardiac, Thoracic and Vascular Sciences, University of Padova, Padova, Italy

[4] Stem Cells & Regenerative Medicine Section, Developmental Biology & Cancer Programme, UCL Great Ormond Street Institute of Child Health, London, United Kingdom

[5] Department of Clinical Genetics, Erasmus Medical Centre, Rotterdam, The Netherlands

* Correspondence: m.pozzobon@irpcds.org; Tel: +30 049 9640126

Academic Editor: name

Received: date; Accepted: date; Published: date

Antibody	Dilution	Manufacturer
Laminin (Rabbit)	1:200	Sigma-Aldrich
αSMA (Mouse)	1:100	Abcam
CD31 (Rat)	1:100	Invitrogen
vWF (Rabbit)	1:200	Dako
CD68 (Rat)	1:100	Abcam
ArgI (Rabbit)	1:50	Thermo Scientific
Anti-GFP 594	1:150	Life Technologies
Anti-Rabbit 488	1:200	Life Technologies
Anti-Rabbit 594	1:200	Life Technologies
Anti-Rat 568	1:200	Life Technologies
Anti-Mouse 488	1:200	Life Technologies
Anti-Mouse 596	1:200	Life Technologies

Table S1: Antibody list

Gene	NM_	FW primer	REV primer	Amplicon (bp)
Flt1	007482.3	AGACCACAGTCTGGCAGTTGG	AGGTTGCCCATGCAGATTCCC	136
Il-6	008656.5	ACAGCAGCTTTGACAGCATC	AAGCAATCCAAGCTGGACAC	85
TNF	001099635.1	AGGCCTTGTGCTTTCCCAGAG	GTTCACAGCATGGTGAACCTGG	86
Nos2	010866.2	CGCTCCAACTGCTCTGATG	TAGTAGGCGGTGTCGTAGCC	86
Arg1	031189.2	GCAATGCACTGGAGTTCG	ACGATGGACGTAAGGGAGTG	94
CCL2	010927.3	GCAGGTCTTTGACGCTCGGA	ATGGCCGACCTGATGTTGCC	105
B2m	009735.3	GCTTCAGTCGTCAGCATGG	CAGTTCAGTATGTTCGGCTTCC	149

Table S2: Primer list

	Native vs DD 7d	DD 7d vs DD 15d	Native vs DD 15d	Native vs ePTFE 7d	ePTFE 7d vs ePTFE 15d	Native vs ePTFE 15d	DD 7d vs ePTFE 7d	DD 15d vs ePTFE 15d	
ADAMTS1	**	**	**	***	**	**	*	**	ADAMTS1
Amphiregulin	***	**	ns	**	*	**	*	*	Amphiregulin
ANG	***	**	***	**	**	**	*	*	ANG
ANGPT-1	*	ns	***	**	***	***	*	*	ANGPT-1
ANGPT-3	**	**	**	***	ns	**	*	**	ANGPT-3
TF	***	**	**	***	**	**	**	*	TF
CXCL16	***	*	**	***	***	**	***	ns	CXCL16
CCN1	ns	*	***	**	**	***	**	***	CCN1
DLL4	*	*	*	***	**	**	***	**	DLL4
CD26	*	ns	**	**	***	***	ns	***	CD26
EGF	*	**	***	**	ns	ns	*	*	EGF
CD105	ns	ns	ns	ns	**	***	ns	**	CD105
Endostatin	**	**	ns	ns	**	***	*	***	Endostatin
ET-1	ns	ns	***	**	*	ns	*	ns	ET-1
FGF-1	ns	**	**	***	***	**	***	**	FGF-1
FGF-2	**	*	*	*	***	***	**	**	FGF-2
KGF	*	*	*	*	ns	ns	**	ns	KGF
Fractalkine	***	**	**	***	***	*	***	ns	Fractalkine
GM-CSF	**	**	***	***	**	**	ns	*	GM-CSF
HB-EGF	**	**	ns	***	***	**	***	**	HB-EGF
HGF	**	*	**	**	*	*	**	ns	HGF
IGFBP-1	***	*	**	*	*	**	*	ns	IGFBP-1
IGFBP-2	**	**	**	**	**	**	**	*	IGFBP-2
IGFBP-3	***	***	*	***	**	**	**	**	IGFBP-3
IL-1a	***	**	**	***	**	**	**	**	IL-1a
IL-1b	**	ns	ns	**	*	**	*	ns	IL-1b
IL-10	ns	ns	ns	***	*	*	*	*	IL-10
CXCL10	**	*	**	***	***	**	***	**	CXCL10
CXCL1	**	*	***	***	**	***	***	**	CXCL1
Leptin	**	ns	*	**	*	**	**	ns	Leptin
CCL2	***	*	**	**	**	***	**	ns	CCL2
CCL3	**	*	*	***	**	*	***	ns	CCL3
MMP-3	***	***	**	**	ns	***	ns	**	MMP-3
MMP-8	**	*	***	***	***	***	**	**	MMP-8
MMP-9	***	**	***	***	*	***	ns	***	MMP-9
NOV	**	**	***	**	*	***	*	***	NOV
OPN	***	***	***	***	ns	***	**	***	OPN
PD-ECGF	**	*	*	ns	ns	*	ns	ns	PD-ECGF
PDGF-AA	ns	**	**	*	ns	ns	*	*	PDGF-AA
PDGF-BB	**	*	*	**	**	*	**	ns	PDGF-BB
PTX-3	**	ns	***	**	**	***	**	*	PTX-3
CXCL4	ns	ns	*	ns	ns	ns	ns	*	CXCL4
PIGF-2	**	*	**	***	**	*	**	ns	PIGF-2
Prolactin	**	ns	***	***	***	**	*	**	Prolactin
Proliferin	**	*	**	***	ns	*	ns	ns	Proliferin
SDF-1	*	ns	ns	***	***	**	***	**	SDF-1
PAI-1	***	***	**	***	***	**	***	*	PAI-1
PEDF	***	***	ns	*	***	**	***	**	PEDF
TSP-2	***	**	***	***	***	***	***	ns	TSP-2
TIMP-1	***	***	**	***	***	***	***	**	TIMP-1
TIMP-4	***	ns	*	**	*	**	*	ns	TIMP-4
VEGF	**	ns	**	**	ns	*	*	*	VEGF
VEGF-B	**	*	*	***	**	**	**	*	VEGF-B

ns P > 0.05 * P ≤ 0.05 ** P ≤ 0.01 *** P ≤ 0.001

Table S3: Statistical analysis
